# Effectiveness of 23-Valent Pneumococcal Polysaccharide Vaccine Against Pneumococcal Diseases Among the Elderly Aged 60 Years or Older: A Matched Test Negative Case-Control Study in Shanghai, China

**DOI:** 10.3389/fpubh.2021.620531

**Published:** 2021-09-20

**Authors:** Xiaodong Sun, Xiang Guo, Jing Qiu, Genming Zhao, Xinxin Xu, Abram L. Wagner, Hongli Jiang, Zhuoying Huang, Jia Ren, Xiaoying Ma, Xiufang Liang, Yao Yao, Jialing Wu, Yihan Lu

**Affiliations:** ^1^Shanghai Municipal Center for Disease Control and Prevention, Shanghai, China; ^2^Department of Epidemiology, School of Public Health, Fudan University, Shanghai, China; ^3^Shanghai Municipal Center for Health Promotion, Shanghai, China; ^4^Department of Epidemiology, School of Public Health, University of Michigan, Ann Arbor, MI, United States; ^5^Department of Health Economics, School of Public Health, Fudan University, Shanghai, China; ^6^Yangpu District Center for Disease Control and Prevention, Shanghai, China; ^7^Chongming District Center for Disease Control and Prevention, Shanghai, China; ^8^Songjiang District Center for Disease Control and Prevention, Shanghai, China

**Keywords:** effectiveness, PPV23, pneumococcal diseases, elderly, test-negative design

## Abstract

**Background:***Streptococcus pneumoniae* infection among adults, especially in adults over 60 years old in China results in a large number of hospitalizations and a substantial financial burden. This study assessed the vaccine effectiveness (VE) of 23-valent pneumococcal polysaccharide vaccine (PPV23) against pneumococcal diseases among the elderly aged 60 years or older in Shanghai, China.

**Methods:** We conducted a test-negative case–control study among the elderly aged 60 years or older who sought care at hospitals in 13 districts of Shanghai from September 14, 2013 to August 31, 2019. A case was defined as pneumococcal disease and testing positive for *Streptococcus pneumoniae*. Controls had symptoms congruent with pneumococcal disease but were negative for *Streptococcus pneumoniae*. We conducted 1:2 matching by gender, age, hospital and admission date. Vaccination status was verified from the immunization system database. VE was calculated with conditional logistic regression according to the formula (1–OR) ×100%.

**Results:** Overall, 603 adults aged 60 years or older with pneumococcal disease and positive for *Streptococcus pneumoniae* were included as cases, and 19.6% (118 persons) had a recorded PPV23 vaccination. The controls included 1,206 adults, whose vaccination rate was 23.8% (287 persons). The VE against pneumococcal diseases among the whole population was 24% (95% *CI*: 2%, 40%) and among women 44% (95% *CI*: 6%, 67%). After adjusting for multiple variables, the effectiveness of PPV23 against pneumococcal diseases was still statistically significant with VE for all of 25% (95% *CI*: 3%, 42%) and VE for women of 49% (95% *CI:* 11%, 71%).

**Conclusion:** PPV23 was effective against pneumococcal diseases in adults aged 60 years or older in Shanghai, China. Its relatively high effectiveness among women warrants this group to be particularly targeted for vaccination, with further research on why vaccination effectiveness is less among men.

## Introduction

*Streptococcus pneumoniae* (*Pneumococcus*) is a common opportunistic pathogen which can colonize the nasopharynx asymptomatically (1). Occasionally, it can invade the lower respiratory tract or the bloodstream and cause various diseases, such as pneumonia, meningitis, bacteremia, otitis media and sinusitis (2, 3). Infants, young children under 2 years old and the elderly are particularly susceptible, along with those with chronic diseases (4–6).

There is a large burden of pneumococcal disease, with 827 thousand deaths from pneumococcal pneumonia and 118 thousand deaths from pneumococcal meningitis worldwide in 2010 (7). There is little information about the burden of pneumococcus in China. It could be responsible for 3.0–8.5% of community-acquired pneumonia in China, although studies in neighboring countries have found larger proportions (e.g., 9.1–65.0% in studies in Japan, and 13.5–60.8% in South Korea) (8).

There are currently two categories of pneumococcal vaccine available in China: a 23-valent polysaccharide vaccine (PPV23) and a 13-valent conjugate vaccine (PCV13). In the US, PPV23 is recommended in all adults ≥65 years old, with PCV13 administered 1 year prior to PPV23 in certain high risk groups (9). Experts within China have also recommended PPV23 vaccination for routine use in adults ≥60 years old (10). A review of PPV23 vaccine studies in older adults have found adequate antibody responses, but mixed results in its effectiveness to prevent illness or death from pneumonia (11). Studies have found PPV23 to be cost-effective within China (12), but these analyses are highly reliant on the estimates of PPV23 effectiveness. In order for there to be a universal recommendation for PPV23 from the Chinese government, more studies of vaccine effectiveness within the country are needed.

Vaccination is an important means of preventing disease and limiting the spread of antibiotic resistance within pneumococcal strains (13). In Shanghai, vaccination of children with PPV23 or PCV13 is not mandatory and requires out-of-pocket payment (14). Since September 14, 2013, Shanghai has launched a major public health service project where residents of 60 years and above can receive a PPV23 vaccination for free. In order to further explore the protective effectiveness of PPV23, a matched test negative case–control study was carried out in Shanghai.

## Methods

### Study Site

This study was conducted in 33 hospitals carrying out the laboratory test project of *Streptococcus pneumoniae* in 13 districts of Shanghai, including Huangpu District, Xuhui District, Putuo District, Jing'an District, Yangpu District, Minhang District, Baoshan District, Jiading District, Songjiang District, Qingpu District, Fengxian District, Pudong New District and Chongming District. Shanghai is one of the most economically developed cities with the highest population density in China, with a population of over 23 million (15).

### Study Population

A matched case-control survey was conducted using the test negative design. We limited our analysis to Shanghai residents aged ≥60 years old because people in this age group are eligible to be given one dose of PPV23 free of charge (16). Patients were eligible to be included if they were hospitalized for diseases congruent with pneumococcal infection, such as pneumonia (including imaging diagnosis of pneumonia), meningitis, bacteremia, otitis media, other lower respiratory tract infections, acute episode of chronic bronchitis, and other lung infection between September 14, 2013 and August 31, 2019. Positive cases were those patients with a positive pneumococcal test by routine culture or PCR test from blood, sputum, cerebrospinal fluid, throat swab, pleural effusion or other samples by urine antigen detection. Those with a negative pneumococcal test from all sources were selected as controls. The final study subjects of the control group were randomly matched by gender (same), age (±5 years), hospital (same) and date of admission (±60 days) in a 1:2 case-control ratio.

### Data Collection

The questionnaire was designed by Shanghai Municipal Center for Disease Control and Prevention (SCDC), and the information of the subjects were retrospectively collected by the project staff who had been trained by SCDC. The investigators collected information related to the patient's demographics (name, gender, age), lifestyle information (smoking status, drinking status), disease history (respiratory related diseases, chronic underlying diseases, immune system diseases), hospitalization information (admission date, antibiotic use, discharge diagnosis, outcomes*), Streptococcus pneumoniae* testing (result of test, samples, and date of sampling), and coinfection (*Mycoplasma pneumoniae, Haemophilus influenzae*, etc.) from different departments in the chosen site hospitals.

Furthermore, we collected vaccination history through the electronic immunization information system maintained by SCDC, which recorded immunization information on all registered residents including date of vaccination, dose and type of vaccine. From this system we abstracted PPV23 and influenza vaccination (within the last year before admission). Subjects without any vaccination records or who received a PPV23 vaccination after discharge were considered as having no PPV23 vaccination.

### Definitions

We determined the types of diseases suffered by the subjects according to ICD codes (International Classification of Diseases, Tenth revision, Clinical Modification) as follows: the ICD codes of pneumonia were J12-J18 and J69, the ICD code for meningitis was G00.1, the ICD code of bacteremia was A40.3, the ICD code of otitis media was H66, the ICD code of other lower respiratory tract infections were J20-22, the ICD codes of acute episode of chronic bronchitis were J44.0 and J44.1, the ICD codes of other lung infection were J98.414 and J98.414A. In addition, in order to include as many cases as possible in the case group, patients with imaging diagnosis of pneumonia (chest radiographs, CT and other ancillary tests indicating pulmonary inflammation) and positive for pneumococcal tests were also included in the case group, which was not applicable in the control group.

### Statistical Analysis

In this test-negative case-control study, the basic characteristics of case and control groups were compared using Chi-square test or Fisher's exact test. Unadjusted and adjusted ORs were estimated using conditional logistic regression models, which included a matching stratum based on gender, age, hospital, and date of admission. We include three sets of models to adjust for possible confounders. Model 1 adjusted for age, medical history (respiratory related diseases, chronic underlying diseases and immune system diseases) and smoking status. Model 2 adjusted for the same inputs as model 1, but also co-infection with other pathogens. Model 3 adjusted for the same inputs as model 2, but also included antibiotics using before sampling and influenza vaccination within previous year.

Vaccine effectiveness (VE) against pneumococcal diseases was estimated as one minus the OR multiplied by 100%. We stratified VE estimates by gender. Statistical analyses were performed in SAS9.4 and two-sided tests were used at a level of 0.05.

### Ethics Statement

This study was approved by the institutional review board of Shanghai Municipal Center for Disease Control and Prevention. We did not obtain informed consent for each study subject because the data were obtained from medical or vaccination registries. Electronic registries included some personal information like name, ID number, cell phone number and address, but these were not included in the datasets made available for analysis.

## Results

**Figure 1:**
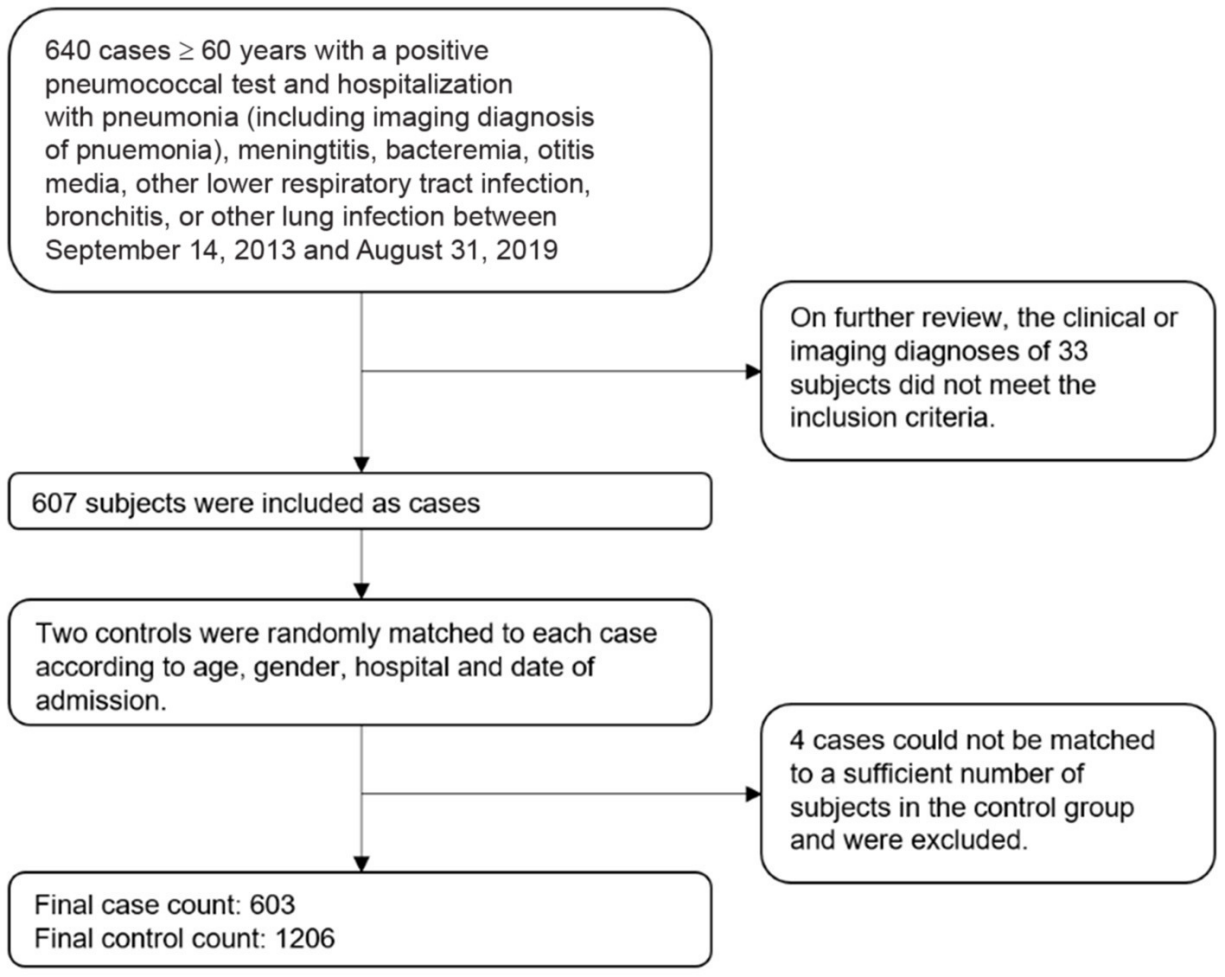
Sampling schematic of cases and controls in the study of pneumococcal vaccine effectiveness in Shanghai.

A total of 640 pneumococcal patients aged 60 years and above with a positive pneumococcal test who were admitted to the investigation site hospitals for pneumococcal diseases during September 14, 2013 and August 31, 2019 were selected into this study as cases (Figure 1). A total of 33 cases were removed after further review indicated they did not meet the inclusion criteria. We removed an additional 4 cases for whom there were not a sufficient number of control subjects to match. The final sample included 603 cases and 1,206 controls.

### Demographic Characteristics

Cases and controls had similar distribution by age, gender, district distribution, drinking status, and history of respiratory related diseases, chronic underlying diseases and immune system diseases, but differed in the other characteristics (Table 1). About 22.1% were women in both of the case group and control group; the ratio of urban and suburban areas in the case group and control group was both about 1:1.8. Of the 603 cases, 492 (81.6%) cases were tested positive in sputum culture, 31 (5.1%) in blood culture, 1 (0.2%) in pleural effusion, 4 (0.7%) in CSF, 4 (0.7%) in throat swab, 46 (7.6%) in urine antigen, 9 (1.5%) in other samples, 8 (1.3%) in both sputum culture and blood culture, 6 (1.0%) in both sputum culture and throat swab, 2 (0.4%) in both sputum culture and urine antigen.

**Table 1:** Basic characteristics of research objects by pneumococcus positive patients (cases) and pneumococcus negative patients (controls).

**Variables**	**Cases (*N* = 603)**	**Controls (*N* = 1,206)**	***P***
**Age group (years old)**			
60–69	190 (31.5)	378 (31.3)	0.991
70–79	159 (26.4)	316 (26.2)	
≥80	254 (42.1)	512 (42.5)	
**Gender**			
Male	470 (77.9)	940 (77.9)	1.000
Female	133 (22.1)	266 (22.1)	
**District^a^**			
Urban	217 (36.0)	434 (36.0)	1.000
Suburban	386 (64.0)	772 (64.0)	
**Smoking**			
Never	345 (57.2)	762 (63.2)	0.006
Quit	101 (16.8)	214 (17.7)	
Yes	138 (22.9)	208 (17.3)	
Unknown	19 (3.2)	22 (1.8)	
**Drinking**			
Never	492 (81.6)	1,012 (83.9)	0.408
Quit	16 (2.7)	38 (3.2)	
Yes	64 (10.6)	105 (8.7)	
Unknown	31 (5.1)	51 (4.2)	
**Respiratory related diseases^b^**			
No	398 (66.0)	831 (68.9)	0.213
Yes	205 (34.0)	375 (31.1)	
**Chronic underlying diseases^c^**			
No	205 (34.0)	370 (30.7)	0.153
Yes	398 (66.0)	836 (69.3)	
**Immune system diseases**			
No	601 (99.7)	1,195 (99.1)	0.168
Yes	2 (0.3)	11 (0.9)	
**Co-infection**			
0 pathogen	328 (54.4)	566 (47.0)	0.001
1 pathogens	116 (19.2)	262 (21.6)	
2 pathogens	67 (11.1)	213 (17.7)	
≥3 pathogens	92 (15.3)	165 (13.7)	
**Antibiotics used before sampling**			
No	176 (29.2)	287 (23.8)	<0.001
Yes	287 (47.6)	792 (65.7)	
Unknown	140 (23.2)	127 (10.5)	
**PPV23 vaccination before hospitalization**			
No	485 (80.4)	919 (76.2)	0.042
Yes	118 (19.6)	287 (23.8)	
**Influenza vaccination within previous year**			
No	603 (100.0)	1,198 (99.3)	0.045
Yes	0 (0.0)	8 (0.7)	

a *Urban districts include Huangpu District, Xuhui District, Putuo District, Jing'an District, Yangpu District, Suburban districts include Minhang District, Baoshan District, Jiading District, Songjiang District, Qingpu District, Fengxian District, Pudong New District and Chongming District*.

b *Respiratory related diseases include chronic respiratory diseases, asthma and pulmonary tuberculosis*.

c *Chronic underlying diseases include hypertension, diabetes, coronary heart disease and chronic liver disease*.

**Figure 2:**
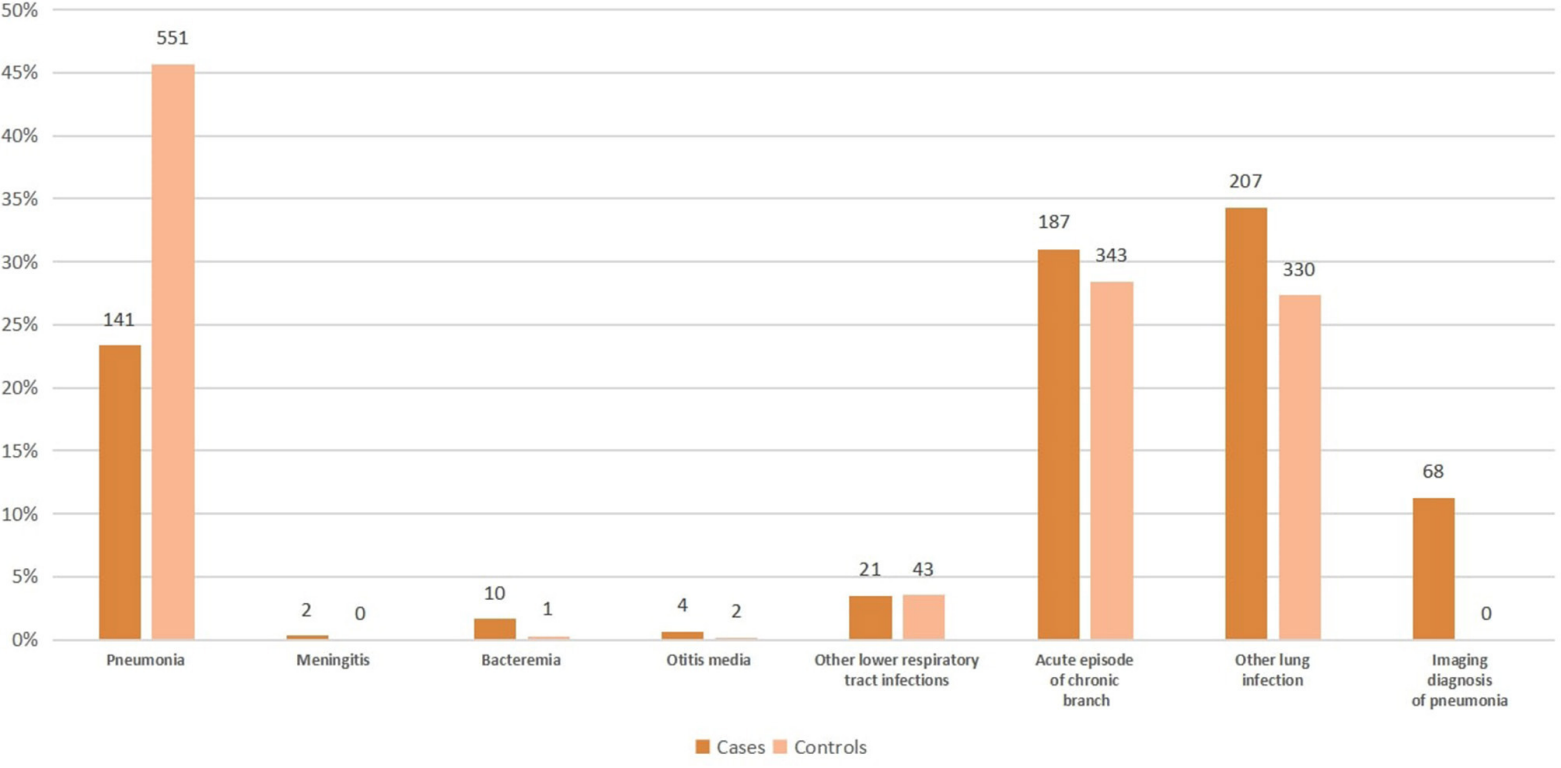
Clinical presentation within cases and controls in the study of pneumococcal vaccine effectiveness in Shanghai.

Figure 2 shows the clinical presentation within cases and controls. A plurality of cases had the other lung infection (34.3%), although acute episode of chronic bronchitis (31.0%) and pneumonia (23.4%) were also common. For controls, a plurality had pneumonia (45.7%).

### Unadjusted VE of PPV23 Vaccination

Through a 1:2 matched analysis, 70 pairs of in the case and control groups were both vaccinated, 753 pairs were not vaccinated in either group, 166 pairs were vaccinated in the case group but not in the control group, while 217 pairs were vaccinated in the control group but not in the case group. The overall unadjusted VE of PPV23 vaccination against pneumococcal diseases was 24% (95% *CI*: 2%, 40%). The point estimate of VE for women (44%, 95%*CI*: 6%,67%) was higher than that for men (16%, 95% *CI*: −12%, 37%) (Table 2).

**Table 2:** Unadjusted VE of PPV23 vaccination against pneumococcal diseases among people aged 60 years or older.

	**a**	**b**	**c**	**d**	**Crude VE, % (95%*CI*)**	***P***
Total	70	166	217	753	24 (2, 40)	0.035
Stratified by gender						
Male	58	130	155	597	16 (−12, 37)	0.236
Female	12	36	62	156	44 (6, 67)	0.030

*^a^Number of pairs that subjects in case and control groups were both vaccinated*.

*^b^Number of pairs that subjects in case group was vaccinated but not in control group*.

*^c^Number of pairs that subjects in control group was vaccinated but not in case group*.

*^d^Number of pairs that subjects in control and case groups were both not vaccinated*.

### Adjusted VE of PPV23 Vaccination

We provide three sets of models to adjust for various confounders, but the estimates of VE remained stable across these models. For model 1, which adjusted for age, medical history (respiratory related diseases, chronic underlying diseases, and immune system diseases) and smoking status, overall VE was 24% (95% *CI:* 3%, 41%), and was lower in men (17%, 95% *CI:* −11%, 37%) than women (47%, 95% *CI:* 9%, 70%). In models 2 and 3, the estimates of VE did not change by more than a few percentage points (Table 3).

**Table 3:** Adjusted VE of PPV23 vaccination against pneumococcal diseases among people aged 60 years or older.

**Multivariable adjustments**	**Adjusted VE,% (95%*CI*)**	***P***
**Overall**		
Model 1	24 (3, 41)	0.030
Model 2	25 (4, 42)	0.025
Model 3	25 (3, 42)	0.030
**Stratified by gender**		
**Male**		
Model 1	17 (−11, 37)	0.215
Model 2	17 (−11, 38)	0.203
Model 3	17 (−11, 38)	0.213
**Female**		
Model 1	47 (9, 70)	0.023
Model 2	50 (13, 72)	0.015
Model 3	49 (11, 71)	0.019

*Model 1: Adjusted for age, medical history (respiratory related diseases, chronic underlying diseases and immune system diseases) and smoking status*.

*Model 2: Adjusted for age, medical history (respiratory related diseases, chronic underlying diseases and immune system diseases), smoking status and co-infection with other pathogens*.

*Model 3: Adjusted for age, medical history (respiratory related diseases, chronic underlying diseases and immune system diseases), smoking status, co-infection with other pathogens, antibiotics using before sampling and influenza vaccination within previous year*.

## Discussion

Widespread pneumococcal vaccination in adults could protect against *Streptococcus pneumoniae* infected diseases. However, there is less information on the effectiveness of the vaccine from observational studies of older adults (17–19). For example, our previous cohort study in Shanghai has found some evidence of a protective effect against community-acquired pneumonia especially among elderly population of lower age (16). But further information about its potential to protect against a wider variety of diseases is needed to support further implementation of pneumococcal vaccination programs in China. In this matched test negative case-control study in China, we examined the effectiveness of PPV23 to protect against pneumococcal disease in older adults, stratified by gender.

This study found PPV23 to be effective in adults aged 60 years or older in Shanghai. Our estimate of vaccine effectiveness is lower than some other studies which examined invasive pneumococcal disease and pneumococcal pneumonia, but our estimate is higher than other studies which examined all-cause pneumonia. For example, the VE of the PPV23 against all-cause pneumonia in a systematic review from 2020 ranged from 3 to 16% (20), overlapping with the results from a systematic review by Kraicer-Melamed et al., where the VE was 17% in cohort studies and 7% in case–control studies in 2016 (21). The VE against pneumococcal pneumonia ranged from 5 to 53% in previous systematic reviews but varied across study designs: VE ranged from 48 to 53% in case–control study, and 37% in a test-negative study reported by Falkenhorst et al. (22), 5–45% in cohort studies reported by Kraicer-Melamed et al. (21) and VE were 45–53% in observational studies by Tin et al. (23). VE against IPD was 29% in a case–control study in South Korea (21); VE against vaccine serotype invasive pneumococcal disease was 42% in the case–control study but 50% in another case–control study, and it was 72.8% with the screening method and 44.5% with the indirect cohort method by Rodriguez et al. (24).

Overall, estimates of VE could vary for a number of reasons. These reasons include how the outcome is defined, serotype distribution, different study designs, characteristics of included participants, time since vaccination, and follow-up time. The range of VE from previous studies highlights the need for local epidemiological work, which can best inform local vaccine policies. The novelty of our study was to look across all pneumococcal disease, ranging from pneumonia, meningitis, bacteremia, and other respiratory infections. We did not find another study which was as comprehensive as ours.

Our study found VE was varied by gender. Similarly, a previous test-negative design study found women have higher protection against pneumococcal pneumonia after PPV23 vaccination in Japan in 2017 (25). Wiemken et al. (26) also reported that effectiveness of PPV23 against hospital admission for pneumococcal pneumonia was higher in women than in men. Reviews of evidence suggest that women are more likely to develop a type III hypersensitivity reaction after PPV23 administration (27), and that serotype-specific pneumococcal antibody concentrations after PCV administration are greater in girls than in boys (28). Our findings support the hypothesis that vaccine effect could be more efficacious in women (29).

## Limitations

Our study has some limitations. Firstly, we used different detection methods (blood culture, sputum culture, sputum PCR, and urinary antigen tests) to identify *Streptococcus pneumoniae* in different hospitals, and the detection limit of each method was different, for example, the PCR-based method can effectively identify pneumococcus in patients who are on antibiotic treatment, but might increase the chance of detection of pneumococcal carriage from respiratory specimens and the use of a low specificity test is known to underestimate true vaccine effectiveness in the test-negative design. The different sensitivities and specificities across these tests could impact our estimates of VE. However, it was difficult to include a sufficient number of cases during the retrospective investigation since no unified standard pneumococcal detection has been carried out in various hospitals. So in order to include more participants in the case group, patients positive for different test methods were included in this study. Furthermore, to include as many cases as possible in the case group, patients with imaging diagnosis of pneumonia (chest radiographs, CT and other ancillary tests indicating pulmonary inflammation) and positive for pneumococcal tests were also included in the case group but not in the control group, which would be a selection bias. Secondly, almost all the studies on PPV23 including the cohort study we conducted before have reported VE stratified by age groups all found decreasing VE with increasing age (20), but this study did not adopt stratified analysis on vaccine effect of different age groups, and also did not perform a stratified analysis of different disease types due to the 1:2 paired design. In addition, we did not analyze the time effect on the relationship between PPV23 vaccination and pneumococcal diseases in our study. Finally, as this was a retrospective case- control study, there was a selection bias in our study, for example: (1) A larger proportion of patients in the control group have pneumonia and other lower respiratory tract infections. (2) In the matching process, the subjects in the control group who have pneumococcal diseases but haven't been tested for pneumococcal test were also matched for the subjects in the case group. However, our study also has some advantages: although it is a case-control study, the recall bias is greatly reduced because the records are directly derived from the hospital information and vaccine information system.

## Conclusion

In summary, the 23-valent pneumococcal polysaccharide vaccine was effective against pneumococcal diseases in adults aged 60 years or older in Shanghai, and it should be recommended, in particularly, for women. These findings can influence future promotion of PPV23 vaccination in other regions in China.

## Data Availability Statement

The raw data supporting the conclusions of this article will be made available by the authors, without undue reservation.

## Author Contributions

XS and XG contributed to the conception and design of the study under the supervision of GZ. XL, JQ, XM, YY, and JW performed the data collection. JQ and XX conducted statistical analysis, wrote the manuscript and revised as needed under the guidance of YL, AW, HJ, XG, and XL. ZH and JR supervised the overall study and provided feedback throughout all stages. All authors contributed to the article and approved the submitted version.

## Funding

Research support funding for this project was received from National Technology Key Research Program of China (2016ZX09101091).

## Conflict of Interest

The authors declare that the research was conducted in the absence of any commercial or financial relationships that could be construed as a potential conflict of interest.

## Publisher's Note

All claims expressed in this article are solely those of the authors and do not necessarily represent those of their affiliated organizations, or those of the publisher, the editors and the reviewers. Any product that may be evaluated in this article, or claim that may be made by its manufacturer, is not guaranteed or endorsed by the publisher.
